# Value of combined detection of serum amyloid A, C-reactive protein and procalcitonin in differential diagnosis of respiratory tract infection in children of China

**DOI:** 10.1080/07853890.2022.2064542

**Published:** 2022-07-01

**Authors:** Hailun Yin, Songming Mo

**Affiliations:** Department of Clinical Laboratory, Tianjin Fifth Central Hospital, Tianjin, China

**Keywords:** Serum amyloid A, C-reactive protein, procalcitonin, respiratory tract infection in children, diagnostics

## Abstract

**Objective:**

To explore the diagnostic value of combined detection of serum amyloid A (SAA), C-reactive protein (CRP) and procalcitonin (PCT) in children with bacteria or non-bacterial respiratory tract infection.

**Methods:**

200 children with respiratory tract infections diagnosed in our hospital were included in the study. According to the results of the aetiological examination, they were divided into bacterial infection group and non-bacterial infection group. At the same time, 100 healthy children admitted to the hospital for physical examination during the same period were selected as the healthy subjects control group. Changes in serum SAA, PCT and CRP in three groups were compared. Comparison of a positive rate of the single index and combined detection were performed. Children with bacterial infections were treated with conventional antibiotics. The changes in serum SAA, PCT and CRP in the infection group before and after treatment were compared. The efficacy of SAA, PCT and CRP alone and in combination was compared.

**Results:**

The serum SAA, PCT and CRP levels in the bacterial infection group were higher than those in the non-bacterial infection group and healthy children, and the differences were statistically significant. The positive detection rates and combined detection rates of serum SAA, PCT and CRP in the bacterial infection group were higher than those in the non-bacterial infection group and the healthy subject's control group. After conventional antibiotic treatment, serum SAA, PCT and CR levels in children with bacterial infection were significantly decreased.

**Conclusion:**

The combined detection based on SAA, CRP and PCT can effectively identify and diagnose respiratory tract infection in children, providing a certain reference for the promotion of the diagnostic scheme.

**Key messages**
- Serum SAA, PCT and CRP were highly expressed in children with respiratory tract infection, and the expression level was the highest in children with bacterial pneumonia.
- The combined detection of serum SAA, CRP and PCT indicators have higher diagnostic efficiency and can effectively make a differential diagnosis of respiratory tract infection in children.

## Introduction

Respiratory tract infections (RTIs) are the leading cause of high mortality in children [1]. Previous studies have shown that the incidence of RTIs in children in China is between 16.8% and 18.7% [2]. The pathogens of this disease are mainly bacteria and viruses. Children are the high-risk population for the disease. Due to age, physiological characteristics and immunity, the disease is harmful to children's health. In addition, as respiratory infections in children are often caused by different pathogens, the lack of typical clinical signs makes the diagnosis of the disease more difficult at an early stage [3]. Studies have shown that more than 80% of children with respiratory tract infections were treated with antibiotics. However, there are many cases of abuse of antibiotics, especially for non-bacterial infections [4]. Therefore, it is very important to determine the pathogen type of respiratory tract infection for the determination of disease treatment. Isolation and culture of pathogens from respiratory tract infections are difficult and time-consuming. Serological indicators are simple, fast, and have a certain value for diagnosis. Erythrocyte sedimentation rate (ESR) and white blood cell (WBC) are commonly used traditional diagnostic indicators, but their defects are low sensitivity and specificity, resulting in a low diagnostic rate of respiratory tract infection in children. In recent years, a number of studies have shown that serum amyloid A (SAA), C-reactive protein (CRP) and procalcitonin (PCT) have certain advantages in the diagnosis of pathogens of respiratory tract infection, which can effectively identify pathogens of infection, improve the diagnostic rate and help to improve the phenomenon of drug abuse [5,6]. Therefore, this study compared SAA, CRP and PCT levels, and analysed the diagnostic value of combined detection based on these three indicators for pathogens of respiratory tract infection in children in China, aiming to provide new ideas for the diagnosis of pathogens of respiratory tract infection in children in China.

## Patients and methods

### Study population and methods

A total of 200 children diagnosed with respiratory tract infection in our hospital from January 2019 to January 2021 were selected as the research participants. Inclusion criteria: (1) Conformity with the Expert Consensus on Standardised Diagnosis and Treatment of Common Colds in Children in China (formulated by Chinese Medical Association) [7]. (2) Age ≤12 years. (3) The case information is true and complete. (4) Subjects and parents informed the study and signed informed consent. Exclusion criteria: (1) Congenital diseases, genetic metabolic diseases or severe primary diseases. (2) 3 days before the study received antibiotics, immunosuppressive agents and other drugs. (3) Antibiotic use taboo. (4) Incomplete personal information. This study was approved by Tianjin Fifth Central Hospital Ethics Committee (WZX-EC-KY2022012). According to the results of the throat swab culture and serum virus test, 200 children were divided into the non-bacterial infection group (100 cases) and bacterial infection group (100 cases).

### Study method

#### Throat swab culture

Patient information is first checked. Use a long cotton swab to gently and quickly wipe the pharynx and tonsils, then insert the swab into the test tube and plug it tightly to prevent contamination of the specimen. After completing the record, label the lab slip to the specimen bottle and send it immediately for an examination.

#### Serum virus test

The gold immunochromatographic assay and immunochromatographic test method was used to detect the serum virus. The threshold value of the serum indicator is the mean A value of the negative sample + 2 times the standard deviation, if this threshold value is reached or exceeded, the test is considered positive.

#### Specimen acquisition

On the day of admission, 1 ml peripheral blood was drawn from children in the non-bacterial infection group and bacterial infection group, and the same amount of peripheral blood was drawn from healthy children on the day of physical examination. 1 ml fasting venous blood was centrifuged at 3500 r/min for 10 min with a centrifugal radius of 8 cm. The upper serum was obtained and placed in a −80 °C refrigerator.

#### Detection of serum PCT, CRP and SAA

PCT detection was completed by Roche electrochemiluminescence analyser (Cobas E601), and original Roche reagents are used. This instrument has high sensitivity (0.005–100 u IU/ml) and specific operation was carried out according to the kit and instrument instructions and the instrument has high. Using the instrument Pumen PA990 and original reagents to complete the detection of CRP level by immune turbidimetry, and the specific operation follows the instructions of the kit machine. The linearity of Pumen PA990 is 2.5–200mg/l. Shenzhen Jinrui PA120 specific protein analyzer was used to detect SAA level; the specific operation follows the instructions. PCT value ≥0.5 ng/mL showed positive reaction. CRP value ≥10mg/L was a positive reaction. SAA value ≥10mg/L showed a positive reaction. In addition, a positive result for one or more of PCT, CRP or SAA is considered positive for the combined detection.

### Outcome measurements

According to the results of pathogen detection, the serum SAA, PCT and CRP levels of children in the three groups were observed and compared. The positive rate of serum SAA, PCT and CRP and the positive rate of combined detection were observed and compared among the three groups. The judgement standard of combined detection was that any positive test of SAA, PCT and CRP was positive. Patients in the bacterial group were treated with antibiotics and the changes in serum SAA, PCT and CRP in the bacterial infection group and non-bacterial infection group were observed and compared after one week of treatment.

### Statistical analysis

Statistical software SPSS26.0 was used to manage and analyse the data. The continuous quantitative data were described by mean ± standard deviation ($\bar{X}\pm S$) or median and range according to normality test results. The mean comparison between groups was analysed by variance analysis, and the pairwise comparison between groups was analysed by the SNK test. If the normality test is not satisfied, then use the non-parametric test. The mean values before and after treatment were compared by correlation sample *t* test. The qualitative data were described in the form of frequency and percentage (%). The *χ*^2^ test was used to compare the rates. The Bonferroni adjustment method was used for pairwise comparison between groups. Except for special instructions, *p* < .05 indicated that the difference was statistically significant.

## Results

### Demographic information of study individual

There were 45 males and 55 females in the non-bacterial infection group, with an average age of 5.42 ± 3.22 years and a course of 2–9 days. There were 56 males and 44 females in the bacterial infection group, with an average age of 5.94 ± 3.56 years and a course of 3–10 days. 100 healthy children who underwent physical examination in our hospital at the same time were selected as the healthy subjects control group. There were 50 males and 50 females, with an average age of 5.61 ± 3.92 years. There was no significant difference in the basic characteristics among the three groups (*p* > .05). See Table 1.

**Table 1.** Demographic information of study individual.

Variables	The non-bacterial infection group	The bacterial infection group	The healthy subjects control group	*P*
Male (%)	45 (45.0)	56 (56.0)	50 (50.0)	>.05
Age (y)	5.42 ± 3.22	5.94 ± 3.56	5.61 ± 3.92	>.05
Course of disease (d)	3.5 (2–9)	4.0 (3–10)	—	>.05

### The results of pathogen types

The results of the tests for the types of bacterial and viral infections were displayed in Table 2. In the bacterial infection group, 45(45.0%) for klebsiella and 9 for streptococcus. In the non-bacterial infection group, 51(51.0%) for influenza A virus and 7(7.0%) for adenovirus. More details were presented in Table 2.

**Table 2.** The results of pathogen types.

Pathogen type	*n*	%
Bacterial type		
Klebsiella	45	45.0
Pseudomonas aeruginosa	21	21.0
Enterobacteriaceae	14	14.0
Staphylococcus	11	11.0
Streptococcus	9	9.0
Virus type		
Influenza A virus	51	51.0
Influenza B virus	19	19.0
Parainfluenza viruses	23	23.0
Adenovirus	7	7.0

### Analysis of SAA, PCT and CRP levels

The differences in serum SAA, PCT and CRP levels among the three groups were statistically significant (*p* < .05). The levels of serum SAA, PCT and CRP in the bacterial infection group were 281.34 ± 42.45, 3.28 ± 1.01 and 42.67 ± 11.02 respectively. Each index of bacterial infection group in the three groups was the highest value. In the pairwise comparison of the three groups, the serum SAA, PCT and CRP levels in the bacterial group were higher than those in the non-bacterial group (*p* < .05), and also higher than those in the healthy subjects control group (*p* < .05). There was a significant difference in serum SAA, PCT and CRP between the non-bacterial group and healthy subjects control group, the non-bacterial group was higher (*p <* .05) (Table 3).

**Table 3.** Comparison of serum SAA, PCT and CRP levels among three groups.

Group	*N*	SAA (mg/L)	PCT (ng/L)	CRP (mg/L)
Bacterial infection group	100	281.34 ± 42.45^ab^	3.28 ± 1.01^ab^	42.67 ± 11.02^ab^
Non-bacterial group	100	41.21 ± 39.87^b^	0.23 ± 0.07^b^	19.79 ± 1.20^b^
Healthy group	100	8.39 ± 3.91	0.08 ± 0.03	5.92 ± 1.95
*F*	—	33.319	15.291	12.417
*P*	—	0.000	0.000	0.001

^a^The difference was statistically significant compared with a non-bacterial group.

^b^The difference was statistically significant compared with the healthy group.

### Comparison of SAA, PCT, CRP positive detection rates and combined detection positive rates

Three groups of single index detection positive rate differences were statistically significant (*p* < .05). The difference between the positive rate of combined detection of three indicators in the three groups was also statistically significant (*p* < .05). The single and combined positive rates of SAA, PCT and CRP in the bacterial infection group were the highest, which were 72.00%, 83.00%, 62.00% and 89.00%, respectively. In the pairwise comparison of the three groups, the positive detection rates of single indicator and combined detection of SAA, PCT and CRP in the bacterial infection group were higher than those in the non-bacterial infection group and the healthy subjects control group, and the differences were statistically significant (*p* < .05). The positive detection rate and combined detection rate of serum SAA, PCT and CRP in the non-bacterial infection group were slightly higher than those in the healthy subjects control group, but the difference between the two groups was not statistically significant (*p >* .05) (Table 4).

**Table 4.** Comparison of SAA, PCT and CRP positive detection rates and combined detection positive rates.

		SAA (mg/L)		PCT (ng/L)		CRP (mg/L)		Combined detection	
Group	*N*	Positive cases	Rate	Positive cases	Rate	Positive cases	Rate	Positive cases	Rate
Bacterial infection group	100	72	72.00^ab^	83	83.00^ab^	62	62.00^ab^	89	89.00^ab^
Non-bacterial group	100	26	26.00	11	11.00	29	29.00	51	51.00
Healthy group	100	13	13.00	4	4.00	15	15.00	29	29.00
$\chi^{2}$		82.454		173.884		78.223		74.909	
*P*		<0.0001		<0.0001		<0.0001		<0.0001	

^a^The difference was statistically significant compared with a non-bacterial group.

^b^The difference was statistically significant compared with the healthy group.

### Comparison of SAA, PCT and CRP before and after treatment in three groups

After one week of conventional antibiotic treatment for children with bacterial infection, the levels of serum SAA, PCT and CRP were significantly lower than those before treatment, and the indicators tended to be normal. The difference before and after treatment was statistically significant (*p* < .05) (Table 5).

**Table 5.** Comparison of serum SAA, PCT and CRP levels before and after treatment in bacterial infection group.

Group	*N*	SAA (mg/L)	PCT (ng/L)	CRP (mg/L)
Before treatment	100	281.34 ± 42.45	3.28 ± 1.01	42.67 ± 11.02
After treatment	100	9.17 ± 2.99	1.07 ± 1.21	4.78 ± 2.11
*t*		59.581	4.128	13.127
*P*	—	<0.05	<0.05	<0.05

## Discussion

Respiratory tract infections are a common respiratory illness, occurring at the turn of the season, especially in children. The clinical features of respiratory infections in children often include cough, runny nose and fever, which if left untreated may prone to develop into lower respiratory tract infection, even causing nephritis, myocarditis, sepsis, rheumatic fever, etc [8]. If the disease cannot be a timely and effective control, the continuous progress of the disease may lead to sepsis, multiple organ failure and septic shock, and even endanger the lives of children [9]. Therefore, timely diagnosis and appropriate treatment are essential for the management of respiratory tract infections. However, conventional culture tests for respiratory tract infections are long, have a low positive diagnosis rate and are of low clinical value. Hence, the search for more accurate and specific tests is of great clinical importance for early and accurate and effective treatment of the disease. In addition, in China, antibiotics are the mainstay of treatment for respiratory tract infections in children. However, antibiotic therapy is not effective for non-bacterial infections and respiratory infections caused by viruses. If antibiotic treatment is applied blindly without a clear diagnosis of the pathogen, it is more likely to increase the risk of dual infection. Therefore, early diagnosis of the disease also plays an important role in determining the type of infection, avoiding the misuse of antibiotics and reducing the incidence of drug resistance.

The results showed that serum SAA, PCT and CRP were highly expressed in children with respiratory tract infection in China, and the expression level was the highest in children with bacterial pneumonia. This has been confirmed in many previous studies [10], suggesting that serum SAA, PCT and CRP are of high value in the differential diagnosis of clinical bacterial respiratory tract infection in children in China. The reasons may be as follows. Serum SAA is often expressed in liver cells and adipocytes, and its concentration will increase by 1000 times in the acute reaction period [11]. Previous studies have compared serum SAA levels in healthy and diseased individuals. The results showed that SAA levels were significantly increased in acute bacterial or viral infections, autoimmune diseases and tumors [12]. Marhaug et al. showed that serum SAA concentration was a very sensitive but non-specific biomarker in the diagnosis, prognosis and monitoring of inflammatory and infectious diseases [13]. In this study, it was found that the serum SAA of children with bacterial respiratory tract infection was significantly higher than that of non-bacterial infection and healthy children, and the difference was statistically significant (*p* < .05). The results were consistent with Chen Jie's results [14]. CRP is one of the important components of the innate immune system. CRP helps clear necrotic or apoptotic cells. CRP began to increase 4 ∼ 6 h after the occurrence of inflammation, doubled every 8 h, reached the peak at 36–50 h, and its half-life was very short. Once the inflammation subsided, CRP would rapidly decrease. At present, CRP is recognised as an effective and intuitive response to the body's sensitive biological indicators, widely used in clinical diagnosis [15,16]. In this study, CRP levels in children with bacterial respiratory tract infection were significantly higher than those in children with non-bacterial respiratory tract infection and healthy children; after conventional antibiotic therapy, CRP levels were significantly reduced to normal levels, indicating that the detection of CRP levels has a guiding role in clinical medication. PCT was first detected in the blood of patients with extrathyroidal diseases in 1993 with a high concentration of calcitonin-like immune reactivity, which was defined as a marker of bacterial infection [17]. Normally, PCT is low in blood circulation (≤ 0.1 ng/mL). Previous studies have shown that PCT has a high diagnostic ability for infection. A large prospective, multicenter study of patients with influenza A (H1NI) showed that PCT < 0.29 ng/mL had a 94% negative predictive value for excluding bacterial co-infection and was superior to CRP [18].

## Conclusion

The combined detection of serum SAA, CRP and PCT indicators have higher diagnostic efficiency and can effectively make the differential diagnosis of respiratory tract infection in children. The combined detection of the three indicators can effectively increase the sensitivity, improve the diagnosis rate of children, and provide a reference for the clinical medication of the disease. It can be used as the basis for the early diagnosis of respiratory tract infection in children and has certain clinical significance.

## Data Availability

All data generated or analysed during this study are included in this published article.

## Abbreviations

SAA: serum amyloid A
CRP: C-reactive protein
PCT: procalcitonin
RTIs: Respiratory tract infections
ESR: Erythrocyte sedimentation rate
WBC: white blood cell
AUC: area under the ROC curve.
